# Review of the Literature on Current Changes in the Timing of Pubertal Development and the Incomplete Forms of Early Puberty

**DOI:** 10.3389/fped.2019.00147

**Published:** 2019-05-08

**Authors:** Giovanni Farello, Carla Altieri, Maristella Cutini, Gabriella Pozzobon, Alberto Verrotti

**Affiliations:** ^1^Pediatric Unit, Department of Life Health and Environmental Sciences, University of L'Aquila, L'Aquila, Italy; ^2^Department of Pediatric, IRCCS San Raffaele Hospital, Milan, Italy; ^3^Pediatric Unit, Department of Biotechnology and Applied Sciences, University of L'Aquila, Aquila, Italy

**Keywords:** puberty, pubarche, telarche, anticipation, development

## Abstract

Puberty is a sensitive period of life characterized by the appearance of secondary sex characteristics which leads to a complete sexual maturation. It physiologically starts between the age of 8 and 13 years in girls and 9 and 14 years in boys. In the last two decades, several studies have showed that start of puberty has moved up to younger ages by 12–18 months, and some of the hypotheses trying to explain this change include the role of nutritional status and obesity and the influence of extrinsic factors such as exposure to endocrine-disrupting chemicals (EDCs), as well. The hypothalamic–hypophysis–gonadal axis develops during embryogenesis, and except for a period of activation immediately after birth, remains suppressed until the onset of pubertal development. At the beginning of puberty, the pulse generator is reactivated, probably due to progressive stimulatory influences on GnRH neurons from glial signals and neurotrasmitters. Kisspeptin and its receptor play a fundamental role in this phase. Premature Pubarche/Adrenarche, Premature Thelarche, and Premature Menarche are incomplete forms of precocious pubertal development that have their origin in endocrine mechanisms that only recently have started to be understood. It is important to distinguish these forms from the complete ones in order to reassure patients and parents about the non-evolution of pubertal progression and avoid non-useful treatments with analogous LHRH.

## Introduction

Puberty is a sensitive period of life characterized by the appearance and gradual development of secondary sex characteristics which leads to complete sexual maturation and reproductive ability (1).

Puberty is not a single event but the completion of a series of maturational steps starting in the uterus and proceeding during the neonatal period; within the first few months of life, the human infant experiences a transient activation of the hypothalamus-pituitary-gonadal axis, (2, 3) a process that has been described as “mini-puberty.” (4) Subsequently, the hypothalamus-pituitary-gonadal axis is inactivated until the beginning of pubertal maturation (5). It physiologically starts between the ages of 8 and 13 years in girls and 9 and 14 years in boys. (6).

Familiar or genetic hereditariness and neuroendocrine factors are some of the determinants of the onset of puberty, which is also influenced by general health, nutrition, exercise and environmental chemicals (7–9).

The completion of puberty is determined by the re-activation of the hypothalamus-pituitary-gonad axis (10) and controlled by neuroendocrine and metabolic factors (11, 12).

The secretion of GnRH is controlled by kisspeptin and its receptor kiss-R1 and is adjusted by neurokinin B and its receptor, whose stimulatory effect increases during puberty, and dynorphin and its receptor, whose inhibitory effect is blocked, resulting in increased GnRH secretion. It has been demonstrated that inactivating mutation in the Kiss-1 or TACR3 genes can result in the absence of puberty (13).

Furthermore, the GnRH pulse source is under excitatory and inhibitory control, so at the onset of puberty the excitatory signal increases while the inhibitory one decreases (14).

The major neurotransmitter responsible for inhibition of GnRH secretion during childhood is gamma amino butyric acid (GABA) while glutamate, neuropeptide Y, endorphins, opioids and melatonin are responsible for activating the GnRH pulse generator and consequently setting up the timing of puberty.

In conclusion, the increased frequency and range of GnRH secretion, along with the increase in excitatory input of kisspeptin through KNDy neurons and glutamate and the decrease in inhibitory signal from GABA neurons, marks the beginning of puberty (5).

Recent studies demonstrated that a switch in the expression patterns of micro-RNAs, which are short non-coding RNAs responsible for silencing gene expression post-transcriptionally, can involve infantile GnRH neurons inverting the balance between inductive and repressive signals, causing an increase in hypothalamic GnRH expression and leading to the start of puberty (15).

Metabolic control is another important factor which influences the onset of puberty, particularly in girls; in fact, important information about the nutritional status and energy reserves are sent to the GnRH neurons (16, 17).

During the peripubertal period there is a change in body composition and sensitivity to insulin, in fact a higher body fat content leads to an earlier pubertal maturation, and early puberty is in turn associated with a higher risk of obesity later in life (18).

On the other hand, puberty is also connected to a decrease in insulin sensitivity (19).

Central adiposity is also associated with decreased insulin sensitivity in women (20), so Hillman et al. tried to evaluate the role of adiposity in the association between early puberty and the changes in insulin sensitivity in school-aged girls, finding that central adiposity is responsible for the majority (more than 75%) of the changes in insulin resistance during early puberty and showing that pubertal maturation is characterized by more than a simple change in secondary sexual characteristics. It is still unclear what the connection between early puberty and obesity in girls is, but some hypotheses support a mismatch between less prenatal weight gain and more postnatal weight gain as a key factor in early pubertal development (21, 22).

In the last two decades, different studies have shown that the start of puberty has moved up in younger ages by 12–18 months (23) and some of the hypotheses conveyed to explain this change include the role of nutritional status and growth but also the influence of extrinsic factors such as the exposure to the endocrine-disrupting chemicals (EDCs) (24). EDCs cause hypomethylation, and potentially should be able to modify the pubertal process and/or the ability of individuals to cope with the environment (25–27). This class of chemicals is capable of interfering with steroid hormone activity, particularly estrogens and antiandrogens as demonstrated in animal models (28) and seems also to be linked to the shift in puberty timing (29).

Variations in the timing of pubertal development are inheritable. This has been demonstrated in the studies on homozygous twins compared to dizygotic twins (30, 31). The knowledge of the underlying mechanisms, including genes that explain variance, is still unclear. Recently, some rare genetic causes of early puberty have been reported and three genes have been identified in the pathogenesis of central precocious puberty: KISS1 (32) encoding kisspeptin, its KISS1R receptor (33) and MKRN3, a gene deemed to act as a hypothalamic repressor on the gonadal axis.

Perry et al. (34) studied 182,416 women from 57 studies, and the authors found 123 signals at 106 genomic loci associated with age at menarche. The genes in the identified loci included those related to the production of GnRH, development and function of the pituitary, bioactivity of hormones, energy homeostasis, growth, and potential peripheral feedback of sex steroids. Some genetic loci identified as influencing pubertal timing had previously been identified as having an impact on BMI, infant growth and adult height (35, 36). The 123 SNPs identified by Perry et al. (37) altogether account for only about 2.7% of menarche age variance, indicating the involvement of multiple genes. Future studies should include interactions with endocrine disruptors that could alter gene expression through epigenetic mechanisms (38)

## Progression of Normal Puberty

The Tanner scale, also known as the Sexual Maturity Rating (SMR) is an objective classification scale used to document and track the development of secondary sexual characteristics in children during puberty.

In females, the normal start of puberty ranges from 8 to 13 years of age, and it is marked by the development of breast buds under the areola; this step is also called thelarche and represents the Tanner Stage B2. In the sequence of events, the thelarche is followed by pubic and axillary hair growth, known as pubarche; in the meantime, growth velocity starts to increase between stage 2 and 3, reaching a peak in stage 4, when the menarche may appear.

In males, the beginning of puberty ranges from 9 to 14 years of age and is marked by a testicular volume equal or >4 ml, representing a Tanner Stage G2. The sequence of events in boys is characterized by the growth of pubic hair and growth of the penis, which follows the enlargement of the testis, and during Tanner stage 3 they reach peak growth velocity, usually 2 years later than girls (39, 40).

## Disorders of Puberty

Over the past centuries, a change in the timing of normal puberty has been demonstrated, with a drastic decline of the age of menarche from 17 years in the early nineteenth century to 13 years in the mid-twentieth century (41). Additionally, the age at the start of breast development seems to be reducing in the last two decades with an average age of the onset of breast development, switching from ~11 years old before the 1980s to the age of 10 years old between 1988 and 1994 (42). The prevalence of PP in girls appears to be about 10 times higher than boys, showing a prevalence in Denmark of 0.2% in girls vs. <0.05% in boys (43).

Otherwise, the secular trend for precocious puberty in the general population is accompanied by an increase in the prevalence and incidence of PP in girls (44).

Some recent American studies have shown unexpectedly early breast development in girls (45), leading Aksglaede et al. to collect new European data to study the timing of puberty by the clinical evaluation of breast development in a cohort of more than 2,000 schoolgirls around Copenhagen and to search for secular trends in the onset of puberty over a 15-year period. What they found out was a decrease in the average age from which glandular breast tissue could be palpated, shifting from 10.9 to 9.9 years among the younger girls living in the same geographical area of the oldest ones. Unusually, they discovered that the early breast development was not connected with a higher level of reproductive hormones, probably suggesting gonadotropin-independent estrogenic actions on the breast instead of an early activation of the pituitary-gonad axis (24). After the evidence of a possible breast development before 8 years of age in American girls, some doubts about the reliability of previous standards have been instilled; a secular shift in age of the start of female puberty has been observed while the age of menarche has changed little over the years. The connection between early puberty and obesity remains controversial while there is partial evidence that an early onset of puberty could be related to hyperinsulinemia and insulin resistance (46).

## Central Precocious Puberty

The estimated incidence of precocious puberty is between 1/5,000 and 1/10,000 (47).

The definition of precocious puberty (PP) in girls is the development of secondary sexual characteristics before the age of 8. The onset of puberty before this age is considered pathological. Because of the obvious anticipation in the age of onset of puberty in some studies, researchers have suggested that age 7 years in girls should be used as a threshold for the classification of precocious puberty (48). Other investigators have subsequently concluded that signs of puberty in girls aged 6–8 years cannot be considered normal or benign as this might lead to underdiagnosed endocrine disorders, so the appropriate threshold for assessment has returned to the previously recommended parameters (49).

Central precocious puberty is caused by early maturation of the hypothalamus-pituitary-gonadal axis and the sexual characteristics are appropriate for the patient's sex (isosexual). The clinical and laboratory evidence of central precocious puberty are reported in Table 1. Central precocious puberty can originate from neurological disorders such as tumors, trauma, or malformations. When there are no detectable CNS lesions, central precocious puberty is defined as idiopathic. These cases might have a genetic, metabolic, or environmental component, or a combination of these factors.

**Table 1:** Clinical and laboratory evidence of precocious puberty.

Development of the breast buds (Tanner B2 or more) in girls before the age of 8 or the enlargement of the testis over 4 ml in boys before the age of 9;
Increased in the growth velocity;
Advanced skeletol maturation;
Enlargement of the size of the ovaries and uterus at the ultrasound examination;
Increased in the basal LH levels and after GnRH stimulation.

Modified from Sultan et al. (5)

Additionally, peripherical production of sex steroids results in gonadotropin-independent precocious puberty. In this form of puberty, sex hormones are usually derived from the gonads or adrenal glands, or from exogenous sources (50).

## Incomplete Forms of Precocious Puberty

In addition to the complete forms of precocious puberty, incomplete forms of precocious and early puberty are present and require a careful, and often difficult, evaluation in order to be able to decide correctly:

- the appropriate investigations;
- the need for a specialist referral;
- the management of treatment.

## Premature Pubarche/Adrenarche

Premature pubarche is a form of incomplete puberty characterized by the presence of pubic hair in girls under 8 years of age and in boys under 9 years of age (51). The functional and morphological changes in the adrenal gland cause an elevation in adrenal androgen precursors causing the clinical manifestation of pubarche/adrenarche. DHA plasma level starts to increase at the age of 6, causing adrenarche (52). With an incidence of 3%, this form of puberty seems to be particularly frequent in the Mediterranean area (11). Additionally, 10–20% of the cases of premature pubarche are associated with insulin-resistance in obese girls (53). Some of the other clinical features are development of axillary hair, acne, oily skin and hair, and adult body odor (5). Skeletal growth and maturation are slightly accelerated, with a good correlation between bone age and chronological age.

It is uncertain if PA is an exaggerated production of DHEA due to early zona reticularis development or an early finding of steroidogenic abnormalities. It is reported that PA increases the risk for PCOS, although it is still not clear if the subjects with exaggerated adrenarche are particularly at risk (54–56).

The causes of premature pubarche/adrenarche have not yet been clarified; one of the theories could be a precocious maturation of the reticularis zone of the adrenal glands, with a peak in the levels of androgens similar to the levels usually reached at the onset of puberty and consequently the growth of pubic hair. In 2006, Vottero et al., tried to search for abnormalities in the androgen receptor (AR) function in both peripheral blood leukocytes and androgen target tissues in girls with premature pubarche and in girls with Tanner stage II; they found out that AR gene methylation was reduced as in girls with Tanner stage II, suggesting an increase in the AR gene activity, therefore leading to a hypersensitivity of the hair follicles to the steroids and to the premature development of pubic hair (57).

The diagnosis is based on the exclusion of other causes such as Precocious Puberty and Non-Classical congenital adrenal hyperplasia (NC-CAH) or a rare virilizing adrenal tumor.

Laboratory assessment for differential diagnosis will measure DHEA, DHEAS, and androstenedione, whose levels may be increased for age in patients with precocious pubarche or similar to the levels normally found in children with Tanner stage II (58); basal serum 17-OH progesterone which may be under 1 ng/ml, followed by an ACTH stimulation test if the 17-OH progesterone is higher than 1 ng/ml.

Basal serum FSH and LH levels after the GnRH stimulation test may be prepubertal, with the left hand and wrist x-ray showing a bone age similar to the biological age (5).

The outcome for girls with premature pubarche is not always positive because the 40% could present a functional hyperandrogenism during adolescence, and these girls have a higher risk to develop a hyperinsulinemia (59). Therefore, a small group of patients with premature pubarche is associated with smallness for gestational age (SGA) (60).

Premature adrenarche is not the exact equivalent for premature pubarche; in fact, it has been recently defined as a condition characterized by an increase in the adrenal androgen levels for the age and sex-specific range associated with an increase in androgen action such as oily hair and skin and adult-type body odor before 8 years of age in girls and 9 years of age in boys (61). Several studies in the past few years have shown an association between this premature androgen excess with low birth weight, metabolic syndrome in children and poly-cystic ovary syndrome (PCOS), suggesting that a pre-natal condition such as low birth weight could be followed by excessive weight gain during childhood, which could lead to a pattern of events culminating with metabolic complications and consequently to premature adrenarche, metabolic syndrome and PCOS (21, 55).

## Premature Thelarche

Premature thelarche is defined as unilateral or bilateral isolated breast development without the development of other sexual characteristics before 8 years of age without the appearance of pubic hair (1). Skeletal maturation is linear, with a good correlation between bone and biological age. In 80% of girls it is possible to find a breast volume between B2 and B3, the breast is often tender, and the palpation can be painful (5).

Some of the possible causes of premature thelarche include increased FSH (but not LH) secretion, excessive dietary intake of estrogens such as phytoestrogens and increased breast sensitivity to circulating estrogens (62), obesity and the role of endocrine-disrupting chemicals (EDCs) such as polybrominated diphenyl ethers (PBDEs), which can cause premature thelarche when it reaches high serum concentrations (63). Crofton et al. tried to test whether premature thelarche is associated with increased FSH-driven follicular development; they measured inhibin B (whose role is stimulating estradiol synthesis) and FSH in girls with premature thelarche, central precocious puberty and normal pubertal controls matched for ages and they found that patients with precocious thelarche had inhibin B and FSH levels much higher than their age-matched controls and similar to the levels found in girls with precocious puberty, providing evidence that precocious thelarche is associated with increased follicular development (64). Laboratory assessment shows increased estrogens levels, increased basal serum FSH levels and, after stimulation with GnRH, nocturnal pulsatility, prepubertal basal serum LH levels and after stimulation with GnRH, possible signs of ovarian follicular development and prepubertal size of the uterus (65). Sometimes precocious thelarche can appear before the age of 2 and, after a variable interval of time, it usually completely regresses; in fact, the more precocious the presentation of thelarche is, the more frequent its regression would be (66). On the contrary, when precocious thelarche appears after the age of 2, especially if there has been more breast development, there will not be a complete regression and, furthermore some of the patients may progress to central precocious puberty (67).

## Premature Menarche

Premature menarche is defined as menstrual-like vaginal bleeding in girls under the age of 9 without the presence of other signs of pubertal development. The differential diagnosis of unexpected vaginal bleeding includes ovarian cysts, infections or tumors of the genital tract, foreign bodies, trauma, sexual abuse, McCune-Albright syndrome or central precocious puberty (68)

It is a rare condition in prepubertal girls which can appear once or be recurring. It usually resolves after 1 or 2 years and is followed by a normal onset of puberty (69). The main cause of this condition is still unknown, but it seems to be associated with a hypersensitivity of the endometrium to very low levels of estrogens, but it does seem to be related to increased gonadotrophins or estradiol levels. Ultrasound examination conducted on these patients also showed a normal prepubertal state of uterine maturation (70).

## Early Puberty

Early puberty is defined by the presence of clinical and auxological signs of pubertal development between the age of 8 and 10 years (71), or between the age of 7.5 and 8.5 years (72) or between the age of 8 and 9 years (73). Some authors found that a relatively early start of puberty may be compensated by a longer duration of pubertal development (74). The mechanism behind early pubertal development has not been clarified yet (75), but some studies have shown an association between restricted prenatal growth and early puberty as a result of a permanent resetting of the endocrine axis (5). This condition may also be related to the secular trend in pubertal timing anticipation (11).

## Conclusions

Although progress has been made in understanding the neuroendocrine mechanisms of puberty development, it is still necessary to clarify the exact mechanisms that underlie it; an increased understanding of puberty regulation will help to improve the treatment of reproductive disorders.

In the last decades we witnessed a stable anticipation of the first steps of pubertal development even though the age of menarche has remained unchanged. Therefore, it is essential to carefully discriminate patients with complete signs of precocity in pubertal development that require appropriate hormone therapies from patients with partial or slow progressive forms in which careful monitoring is acceptable.

This is even more important given the availability of effective hormonal treatments (analog LHRH) able to block the progression of puberty development in cases of true and complete precocity.

For this purpose, it is necessary to be aware of the incomplete and non-progressive forms of precocious puberty that we find, more and more frequently, in clinical practice given the improvement of living conditions, the increase in the incidence of overweight and obesity and the presence of “disruption of chemicals” in the environment and in food, which seems to be able to induce progressive anticipation of the onset of puberty.

A limitation in our review is the impossibility of specifying the exact role of endocrine disruptors in the development of sexual precociousness, because the available studies are still preliminary and do not reach an univocal conclusion. While confirming the presence of inheritance in advances of pubertal development, more large-scale studies are needed to establish with certainty which genes can be involved in the different forms of anticipation of puberty development.

## Author Contributions

GF and GP substantial contributions to the conception or design of the work. MC and CA drafting the work or revising it critically for important intellectual content. AV wrote the first draft of the manuscript. All authors contributed to manuscript revision, read and approved the submitted version.

### Conflict of Interest Statement

The authors declare that the research was conducted in the absence of any commercial or financial relationships that could be construed as a potential conflict of interest.
